# HIV care and treatment clinic performance following President’s Emergency Plan for AIDS Relief-funded infrastructure improvement in Tanzania

**DOI:** 10.4102/sajhivmed.v19i1.777

**Published:** 2018-06-14

**Authors:** Boniphace M. Idindili, Simon J. King, Kristen Stolka, Irene Mashasi, Philberth Bashosho, Happy Karungula, Florida Chintowa, Godfrey Mwakabole, Kimberly Ashburn, Barbara Do, Norman Goco

**Affiliations:** 1 RTI International, Tanzania; 2 RTI International, United States; 3 Independent consultant, Tanzania; 4 Center for Applied Public Health Research, RTI International, United States

## Abstract

**Purpose:**

To assess how the infrastructure improvements supported by the US Centers for Disease Control and Prevention (CDC) and the United States President’s Emergency Plan for AIDS Relief (PEPFAR) contributed to facility-level quarterly and annual new patient enrolment in HIV care and treatment and antiretroviral therapy (ART) uptake and retention in care.

**Methods:**

Aggregate quarterly and annual facility-based HIV care and treatment data from the CDC-managed PEPFAR Reporting Online and Management Information System database collected between 2005 and 2012 were analysed for the 11 rural and 32 urban facilities that met the eligibility criteria. Infrastructure improvements, including both renovations and new construction, occurred on different dates for the facilities; therefore, data were adjusted such that pre- and post-infrastructure improvements were aligned and date-time was ignored. The analysis calculated the mean (95% confidence interval) number of patients per facility who were (1) newly enrolled in HIV care, (2) patients initiated on ART, (3) patients retained in care, defined as alive and on ART, and (4) reasons for attrition, defined as transferred out, lost to follow-up, deceased or stopped ART.

**Results:**

The overall mean number of adult patients newly enrolled in HIV care clinics per quarter declined from 187.7 (151.4–223.9) to 135.2 (117.4–152.9) after infrastructure improvements but was not statistically significant (*p* = 0.20). However, the mean number of patients who were alive and remained on ART increased from 193.2 (145.3–241.1) to 273.2 (219.0–327.3) after improvements in both rural and urban facilities, although not significantly (*p* = 0.59). A similar picture was observed for overall paediatric enrolment and retention in care. Health facility-specific case studies show variations in new patient enrolment and retention in care between health facilities depending on the catchment area, population HIV prevalence and coverage of ART facilities. Regarding attrition, the mean number of adult patients lost to follow-up changed from 76.6 (20.8–132.3) to 139.4 (79.6–199.1) (*p* = 0.65) among rural facilities, while the mean number of children lost to follow-up increased significantly from 3.4 (0.5–6.3) to 8.7 (5.0–12.3) (*p* = 0.02) after improvements.

**Conclusion:**

Patient retention in care improved in HIV care and treatment facilities with infrastructure improvements. However, the overall number of patients newly enrolled and initiated on ART declined and attrition increased in facilities after improvements.

## Background

HIV/AIDS morbidity and mortality has overburdened the health system in Tanzania and other countries in sub-Saharan Africa for over three decades without effective interventions.^1,2,3^ Because of the high burden of people living with HIV (PLHIV) in the early 2000s, Tanzania experienced a high demand for HIV care and treatment services but faced challenges such as limited budget for health services, poor infrastructure, shortage of health workers and a fragmented procurement and supply system.^4,5^ Antiretroviral therapy (ART) services were provided by a few private hospitals at a price that could not be afforded by the majority of HIV-infected patients. By December 2004, only 3000 HIV patients in Tanzania were reported to be receiving ART, while the estimated demand at that time was 440 000 patients.^2,6^

Tanzania began providing free access to ART in September 2004 under the HIV/AIDS Treatment and Care Plan 2003–2008.^6^ HIV/AIDS care and treatment centres were initially established in four referral hospitals and five regional hospitals. Antiretroviral (ARV) users travelled long distances and spent a significant amount of money for monthly travel to clinics to seek HIV care and treatment.^7^ Initially, the majority of HIV-positive persons were found in urban areas where the initial HIV epidemic in Tanzania was identified. Over time, as the epidemic spread to rural areas, the existing health system was unable to meet the ART demands of the increased number of people living with HIV/AIDS.^8^

UNAIDS and WHO released the 3 by 5 Strategy in 2003, which aimed at mobilising international donor agencies to work together to assist developing countries to fight the HIV/AIDS pandemic.^9^ Tanzania received funding for HIV/AIDS care and treatment scale-up from organisations such as the Global Fund to Fight AIDS, Tuberculosis and Malaria (the Global Fund), the US President’s Emergency Plan for AIDS Relief (PEPFAR), the Clinton Foundation, the Swedish International Development Agency and the Tanzania Multicountry HIV/AIDS Programme.^3,10^

PEPFAR, through the US Centers for Disease Control and Prevention (CDC), provided financial and technical support to Tanzania in the rapid scale-up of HIV/AIDS prevention, care and treatment programmes, health system strengthening and impact mitigation. As part of health system strengthening, PEPFAR invested approximately $33 million for renovation and construction of laboratory and HIV care and treatment clinic buildings between 2005 and 2012 in 132 sites across Tanzania.^11,12^ The infrastructure improvements improved work space, accommodated additional services and improved patient flow to access HIV-related services.

Through PEPFAR support, Tanzania made tremendous achievements in the fight against HIV/AIDS. HIV prevalence decreased from 7.1% in 2001 to 5.0% in 2011 in adults 15–49 years (Tanzania Health and Malaria Indicator Survey [THMIS] 2011–12). More than 1.1 million pregnant women received HIV testing and counselling. The number of clients who received HIV testing and counselling results was 105 000 in 2004 and increased to 3.37 million in 2012. During the same period, the number of patients on ART increased from 1518 to 364 000^11^ and the number of HIV/AIDS care and treatment clinics increased from 22 to 729.

### Rationale

Increased funding from the Global Fund and PEPFAR as well as competition from generic manufacturers drastically improved availability of ARVs in developing countries, including Tanzania.^13^ As a result, PLHIV overcrowded the few available care and treatment centres. Health facilities accredited to provide ART services experienced structural problems with potential impact on patients’ access and retention in care. Most ART services were provided in temporary shelters or a single shared room that lacked both confidentiality and privacy, leading to poor quality of care, stigma, and discrimination.^7^ In addition, poor laboratory services to support patient care were common in almost all HIV care and treatment facilities.^12^

This study was designed to evaluate the impact of infrastructure investments to support the scale-up of ART on the delivery of services, particularly in terms of patient enrolment and uptake of services and retention in care. The results of this evaluation will provide information for future investments in health facility infrastructure improvement.

Although PEPFAR has invested in a range of HIV intervention programmes, the contribution of physical infrastructure improvements on patient outcomes in line with the scaling up of ART is not documented. Infrastructure improvement inputs are postulated to affect the quality of the care environment by providing adequate physical space to offer privacy for confidential patient counselling and examinations, enhanced access to and organisation of patient records, and increased confidence in treatments offered to clients.^14^ Investments in physical buildings may contribute to the efficient use of space as planned for HIV services and possibly for additional HIV or non-HIV services as well. Improving infrastructure enhances how patients are able to access services, service utilisation, staff job satisfaction and patient satisfaction with services. We acknowledge that other individual factors contribute to enrolment and retention to care, such as transportation to clinics, financial constraints, social support and stigma, and perception of ‘feeling well’.^15^ However, we hypothesised that investments in infrastructure improvements were expected to lead to increased numbers of patients coming to the facility for services and ultimately the increased enrolment and retention in HIV care and treatment patients.

## Methods

### Data collection and reporting

This infrastructure evaluation was designed as a longitudinal, retrospective evaluation of PEPFAR-funded, CDC-managed infrastructure improvements completed in selected facilities in mainland Tanzania. Among 244 PEPFAR-supported infrastructure improvement sites located in 22 regions of Tanzania, we selected 86 health facilities using census sampling using the following eligibility criteria: CDC-managed, US Department of State Regional Procurement Service-funded investments in infrastructure in mainland Tanzania during 2007–2009 and new construction or renovation fully completed (officially handed over from CDC-managed contract to the responsible partner or agency). These facilities are located in rural and urban settings and range from health centres providing primary care to regional hospitals providing tertiary care.

Tanzania uses the PEPFAR Records Organization Management Information System (PROMIS) database to collect and aggregate quarterly report data from health facilities providing HIV care and treatment, as well as community-based programmes. This database was established in 2006 to track facility-level data from all CDC-supported HIV care and treatment facilities.

At the facility level, patients obtaining HIV/AIDS care and treatment services are registered in the attendance register; demographic and clinical characteristics and treatment information are recorded on the patient medical card. The data are then entered into a Ministry of Health Microsoft Access database, which is maintained at the facility, updated with every patient visit and used for reporting to the national government. The non-governmental organisation providing technical support to the HIV care and treatment facility; Ministry of Health, Community Development, Gender, Elderly and Children; and the Regional and District Health Management Teams provides regular supportive supervision to the facility-level data entry clerks for quality assurance. The non-governmental organisation implementing partner available at the facility is also responsible for aggregating the facility-level data into quarterly data reports and submitting them to the CDC PROMIS database in the facility-based HIV Care and ART reporting form. Quarterly data submitted to PROMIS between January 2007 and June 2012 were used for this analysis.

### Data analysis

Time points are presented as quarters before and after infrastructure improvements. The date of completion of infrastructure improvements is considered time point zero. Two study periods were analysed: (1) the period of at least four quarters *before* the improvement of the HIV care and treatment building and (2) the period of at least four quarters *after* commissioning the improved building for use. In defining the quarters, it was critical to ensure the following:

- Clinics were reporting during both time points.
- Clinics were reporting the indicator of interest during both time points.

This process was repeated for all analyses, including subpopulation analyses (e.g. urban vs. rural), and led to the inclusion of 43 health facilities (11 rural and 32 urban) for final analysis. The main outcomes of interest included patients newly enrolled in HIV care; patients initiated on ART; patients retained in care, defined as alive and on ART; and reasons for attrition, defined as transferred out, lost to follow-up, deceased or stopped ART. For each outcome of interest, the mean (95% confidence interval) number of patients was calculated for the four quarters before and four quarters after infrastructure improvements using STATA version 12. Students’ *t*-test and corresponding *p*-values were calculated for the comparison of the before and after means. Some indicators are distributed normally, some uniform, some skewed. There is no perfect measure of central tendency to cover this range of score distributions, but because the assumptions of the central limit theorem were not violated, it was decided to use the mean. Additionally, a pre-post difference was desired.

In addition, we analysed patient data at facility level to present trends in patient enrolment and retention in care before and after improvements. These analyses are presented as health facility-specific case studies.

## Ethical consideration

The study received ethical clearance from the Tanzania Medical Research Coordinating Committee of the National Institute for Medical Research and CDC Center for Global Health Associate Director for Science.

## Results

We discuss changes in key indicators before and after infrastructure improvements. Overall results are presented by adult (Table 1) and child (Table 2) patient outcomes, by sex and by location (rural or urban). Key indicators are presented in the following order: (1) patients newly enrolled in HIV care and treatment, (2) patients newly initiated on ART, (3) patients retained in care (alive and on ART) and (4) reasons for attrition such as transferred out, lost to follow-up, deceased or stopped ART. These overall results are complemented by health facility-specific case studies and graphs to show the variation in trends in patient outcomes observed at individual health facilities before and after infrastructure improvements.

**TABLE 1:** Mean numbers of HIV-positive adults newly enrolled in care, initiated on antiretroviral therapy and retained in care before and after construction of HIV buildings in selected health facilities.

Facility location and outcome indicator	Before construction			After construction			*p*
	Number of facilities	Mean number of patients	95% confidence interval	Number of facilities	Mean number of patients	95% confidence interval	
**New enrolment to care**	**43**	**187.7**	**151.4–223.9**	**43**	**135.2**	**117.4–152.9**	**0.20**
Rural facilities	11	62.6	45.0–0.2	11	53.6	39.1–68.1	0.62
Male	11	22.4	15.4–29.5	11	21.2	14.6–27.8	0.80
Female	11	40.1	29.4–50.8	11	32.4	24.4–40.4	0.25
Urban facilities	32	230.7	184.5–276.8	32	163.2	141.9–184.6	0.12
Male	32	81.5	63.9–99.1	32	58.9	50.7–67.1	0.02*
Female	32	149.2	120.3–178.0	32	104.3	90.8–117.8	0.01*
**New on ART**	**43**	**90.2**	**72.5–107.9**	**43**	**66.0**	**57.7–74.4**	**0.37**
Rural facilities	11	33.8	22.2–45.3	11	26.9	20.6–33.3	0.99
Male	11	12.8	8.2–17.3	11	10.6	7.72–13.6	0.43
Female	11	21.0	13.8–28.1	11	12.7	12.7–19.9	0.24
Urban facilities	32	109.6	87.1–132.2	32	79.5	69.4–89.5	0.30
Male	32	39.2	30.8–47.5	32	28.9	24.9–32.9	0.03*
Female	32	70.5	56.1–84.8	32	44.1	44.1–56.8	0.01*
**Retained in care (Rural facilities)**							
Alive and on ART	11	193.2	145.3–241.1	11	273.2	219.0–327.3	0.59
Transferred out	5	17.4	5.9–29.0	5	61.9	33.6–90.2	0.50
Lost to follow-up	6	76.6	20.8–132.3	6	139.4	79.6–199.1	0.65
Deceased	6	28.4	15.4–41.3	6	48.7	27.7–69.6	0.70
Stopped ART	5	3.6	1.1–6.2	5	4.8	2.7–7.0	0.49
**Retained in care (Urban facilities)**							
Alive and on ART	32	668.9	560.1–777.7	32	895.9	771.7–1020.0	0.64
Transferred out	18	163.9	108.4–219.4	18	315.3	235.5–395.2	0.28
Lost to follow-up	17	207.7	147.4–267.9	17	325.8	258.5–393.1	0.94
Deceased	17	93.6	79.9–107.3	17	150.2	127.3–173.1	0.50
Stopped ART	15	7.5	5.7–9.3	15	10.0	7.0–13.0	0.52

ART, antiretroviral therapy.

* *p* < 0.05

**TABLE 2:** Mean numbers of HIV-positive children newly enrolled in care, initiated on antiretroviral therapy and retained in care before and after construction of HIV buildings in selected health facilities.

Facility location and outcome indicator	Before construction			After construction			*p*
	Number of facilities	Mean number of patients	95% confidence interval	Number of facilities	Mean number of patients	95% confidence interval	
**New enrolment to care**	**43**	**16.4**	**13.1–19.8**	**43**	**11.3**	**9.8–12.8**	0.01*
Rural facilities	11	6.8	4.1–9.4	11	5.6	4.3–6.9	0.44
Male	11	3.3	2.1–4.6	11	3.1	2.3–3.8	0.73
Female	11	3.5	1.9–5.0	11	2.6	1.7–3.4	0.29
Urban facilities	32	19.8	15.5–23.9	32	13.3	11.5–15.1	0.01*
Male	32	9.5	7.4–11.5	32	6.3	5.4–7.3	0.01*
Female	32	10.3	8.0–12.6	32	6.9	5.9–7.9	0.01*
**New on ART**	**43**	**6.7**	**5.3–8.2**	**43**	**5.9**	**5.1–6.8**	**0.34**
Rural facilities	11	3.2	1.0–5.3	11	3.2	2.0–4.3	0.99
Male	11	1.7	0.6–2.7	11	1.6	1.1–2.2	0.97
Female	44	1.5	0.4–2.6	11	0.7	0.8–2.2	0.97
Urban facilities	32	8.0	6.3–9.8	32	6.9	5.8–8.0	0.30
Male	32	4.1	3.1–5.0	32	3.3	2.7–3.9	0.18
Female	32	3.9	3.1–4.8	32	3.0	3.0–4.2	0.54
**Retained in care (Rural facilities)**							
Alive and on ART	11	17.5	13.0–22.0	11	27.5	21.5–33.5	0.01*
Transferred out	5	2.2	1.1–3.4	5	5.8	3.1–8.5	0.02*
Lost to follow-up	6	3.4	0.5–6.3	6	8.7	5.0–12.3	0.02*
Deceased	6	1.7	0.6– 2.9	6	3.1	1.5–4.7	0.18
Stopped ART	5	0.1	0.0–0.3	5	0.6	0.2–0.9	0.03*
**Retained in care (Urban facilities)**							
Alive and on ART	32	55.4	44.4–66.3	32	72.3	60.0–84.6	0.04*
Transferred out	18	11.6	7.5–15.6	18	22.7	16.5–28.8	0.003*
Lost to follow-up	17	12.7	9.2–16.4	17	22.7	17.0–28.3	0.004*
Deceased	17	7.9	5.9–9.7	17	14.3	10.6–17.9	0.002*
Stopped ART	15	0.4	0.2–0.5	15	0.5	0.2–0.7	0.56

ART, antiretroviral therapy.

* *p* < 0.05

### New enrolment to care

The overall mean number of adult patients newly enrolled in HIV care per quarter declined from 187.7 (151.4–223.9) to 135.2 (117.4–152.9) patients after infrastructure improvements but was not statistically significant (*p* = 0.20). However, the urban facilities experienced a significant decrease in the quarterly mean number of patients for both females and males newly enrolled in care after improvements (before: males 81.5 [63.9–99.1], females 149.2 [120.3–178.0]; after: males 58.9 [50.7–67.1], females 104.3 [90.8–117.8]; *p* = 0.02 and *p* = 0.01, respectively) (Table 1).

The overall mean number of children newly enrolled in HIV care per quarter declined significantly from 16.4 (13.1–19.8) to 11.3 (9.8–12.8) after improvements (*p* = 0.01). This significant drop was seen primarily in urban facilities (before: 19.8 [15.5–23.9]; after: 13.3 [11.5–15.1], *p* = 0.01) (Table 2).

#### Newly initiated on antiretroviral therapy

The mean number of adult patients newly initiated on ART saw an overall decrease after improvements. For the rural health facilities, the mean changed non-significantly from 33.8 (22.2–45.3) before improvements to 26.9 (20.6–33.3) after improvements (*p* = 0.99). However, there was a significant drop among both males and females after improvements in urban health facilities (*p* = 0.03 and *p* = 0.01, respectively) (Table 1).

Overall, the mean number of children newly initiated on ART per quarter did not change significantly among rural and urban facilities after improvements (*p* = 0.34). Rural facilities reported no change in the mean number of children who started on ART after improvements while urban health facilities recorded a non-significant drop (before: 8.0 [6.3–9.8]; after: 6.9 [5.8–8.0], *p* = 0.30) (Table 2).

**FIGURE 1:**
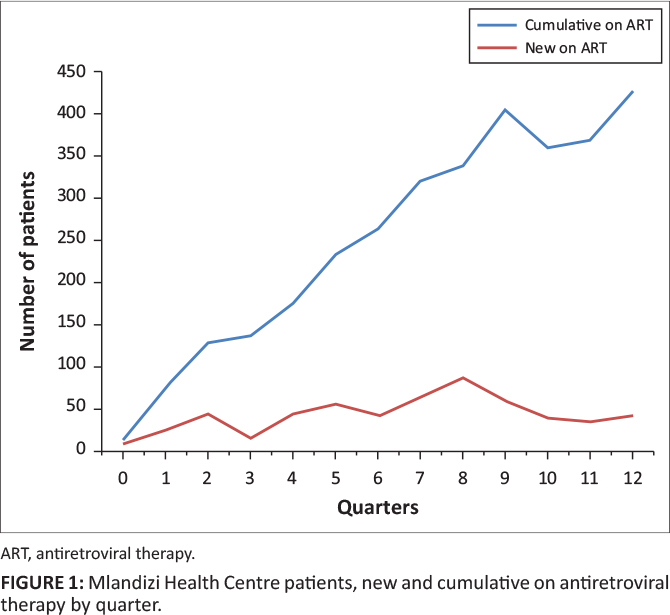
Mlandizi Health Centre patients, new and cumulative on antiretroviral therapy by quarter.

**FIGURE 2:**
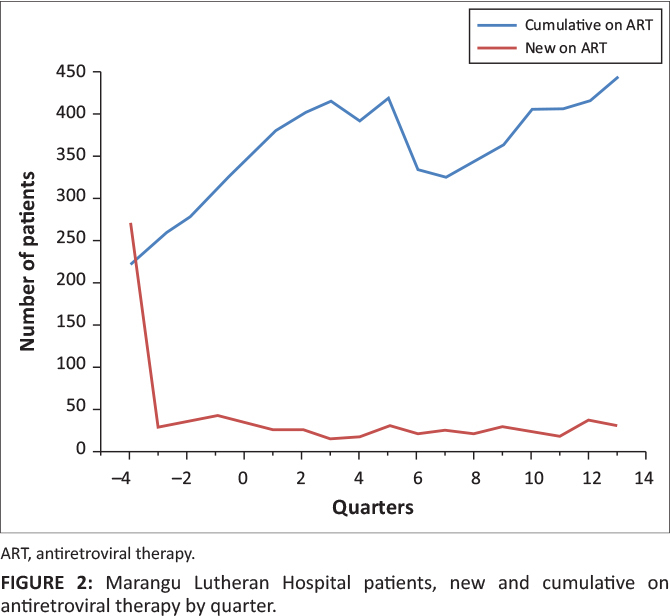
Marangu Lutheran Hospital patients, new and cumulative on antiretroviral therapy by quarter.

**FIGURE 3:**
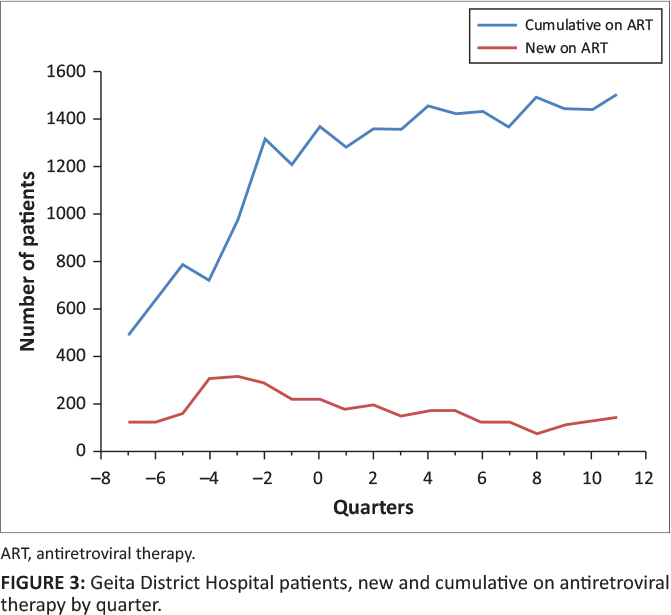
Geita District Hospital patients, new and cumulative on antiretroviral therapy by quarter.

### Retention in care

Overall retention in care did not change significantly for adult patients who were alive and on ART in both rural and urban facilities. For the 11 rural facilities that reported for the four quarters before and after improvements, the mean number of patients retained in care changed from 193.2 (145.3–241.1) to 273.2 (219.0–327.3) after improvements. At the same time, for the 32 urban facilities, the mean number of patients alive and on ART increased from 668.9 (560.1–777.7) to 895.9 (771.7–1020.0) after improvements. However, these differences were not statistically significant (*p* = 0.59 and *p* = 0.64, respectively) (Table 1).

On the other hand, the mean number of children retained in care increased significantly across both rural and urban health facilities. In rural health facilities, the mean number of children retained in care and active on ART increased significantly from 17.5 (13.0–22.0) to 27.5 (21.5–33.5) after improvements (*p* = 0.01). Urban facilities saw an increase from 55.4 (44.4–66.3) to 72.3 (60.0–84.6) (*p* = 0.04) (Table 2).

### Attrition

Attrition at each facility occurred because of a number of factors including transfers, loss to follow-up, death and stopping ART. Loss to follow-up accounted for the majority of attrition during this study period. Loss to follow-up is defined as patients who do not return to care after a specified period of time because of a variety of reasons, which may include social and economic factors such as lack of transport or stigma, transfer to another clinic or voluntarily dropping out of care.^16^ This evaluation did not analyse factors associated with retention in care or attrition in this study population. For the six rural facilities that had complete data for all four quarters before and after improvements, the mean number of patients lost to follow-up changed non-significantly from 76.6 (20.8–132.3) to 139.4 (79.6–199.1) (*p* = 0.65). For the 17 urban facilities, the mean number of patients lost to follow-up changed from 207.7 (147.4–267.9) to 325.8 (258.5–393.1) (*p* = 0.94). Death comprised the next largest reported contribution to adult attrition; the mean number of patient deaths for the rural facilities was 28.4 (15.4–41.3) before and 48.7 (27.7–69.6) after improvements, a non-significant increase across six facilities (*p* = 0.70). The mean number of adult patient deaths for 17 urban facilities increased non-significantly from 93.6 (79.9–107.3) to 150.2 (127.3–173.1) (*p* = 0.50) (Table 1).

For children, loss to follow-up was the leading cause of attrition. The mean number of children lost to follow-up increased significantly in both rural and urban facilities (*p* = 0.02 and *p* = 0.004, respectively). The second largest reported contribution to attrition among children was transferring to another facility. The mean number of children who transferred out of rural facilities significantly increased from 2.2 (1.1–3.4) to 5.8 (3.1–8.5) after improvements (*p* = 0.02). Among urban facilities, the mean number of transfers also increased significantly from 11.6 (7.5–15.6) to 22.7 (16.5–28.8) (*p* = 0.003). In addition, there was a notable increase in deaths among children in urban facilities (before: 7.9 [5.9–9.7]; after: 14.3 [10.6–17.9], *p* = 0.002) (Table 2).

### Impact of infrastructure improvements on performance of health facilities: Case studies

As a complement to overall results, we present case studies that show health facility-specific variations in trends of patients newly enrolled in care and initiated on ART and cumulative number of patients on ART, before and after infrastructure improvements. Case studies include graphs that show the trend in patient outcomes before and after improvements by quarter. Time point zero on the *x*-axis of the graph indicates the time point at which infrastructure improvements were completed.

**Case study 1:** Mlandizi Health Centre experienced an increase in patients newly enrolled and initiated on ART. The health centre started providing ART services in November 2008 immediately after construction of the HIV care and treatment building. Prior to construction of the Mlandizi HIV care and treatment clinic, patients had to travel about 40 km to access ART care and treatment services. The graph shows that over time there have been progressive increases in the number of patients newly initiated in ART and the cumulative number of patients currently on ART. The presence of an HIV clinic within the community reduced the travel distance and time to receive services, as well as transport costs for patients.

**Case study 2:** Marangu Lutheran Hospital experienced a decrease in the number of newly enrolled patients but a steady increase in cumulative patients on ART after improvements. At the beginning of the provision of HIV services in 2007, four quarters prior to construction, there were more than 250 newly enrolled patients on ART. This was followed by a sharp drop in enrolment of new patients on ART to fewer than 50 patients per quarter, which remained steady even after construction. On the other hand, we observed a progressive increase in cumulative number of patients who remained alive and on ART after construction.

**Case study 3:** Geita District Hospital experienced a decrease in enrolment after infrastructure improvements but a steady rise in cumulative patients on ART. The decline in enrolment of new patients persisted consistently for more than eight quarters after improvements. The decline can be explained by the effects of establishing new ART sites in health centres within the district catchment area. The cumulative number of patients on ART continued to increase, which was likely because of the transfer of patients from other clinics.

## Discussion

The construction and renovation of HIV care and treatment centres took place during the scale-up of ART services in Tanzania. HIV care and treatment services were decentralised to lower level health facilities from the initial urban higher level health facilities.^17^ After infrastructure improvements, the new clinics in rural and peri-urban settings reduced the work load of health workers in urban health facilities.

The trends of both adults and children newly enrolled to HIV care and initiated on ART show mostly non-significant declines in health facilities after infrastructure improvements. Significant declines in new enrolments to care and initiation to ART were seen in urban facilities, which we suspect is because of patients enrolling at rural facilities now closer to their homes. For children, this general trend of declines is surprising because of the contribution of prevention of mother-to-child transmission (PMTCT) programmes in child entry to HIV care and treatment during the post-infrastructure improvement study period.^18,19^ PMTCT programme efforts were intensified in the country following PEPFAR funding and have increased pregnant women’s access to ART and linking children into HIV care and treatment. It is possible that the successful implementation of PMTCT programmes during pregnancy and delivery have reduced the incidence of HIV among infants at birth.

Our analysis showed an overall decline in patients newly enrolled in HIV care and initiated on ART. However, health facility-specific case studies demonstrate both positive and negative trends observed in enrolment of new HIV patients and ART initiation following infrastructure improvements. The infrastructure improvements established new HIV care and treatment centres with the aim of increasing coverage and access to ART but also coincided with additional training of health workers, improved laboratory services and establishment of new HIV laboratories in lower level health facilities together with ART initiation centres. Depending on its location, a health facility in a high HIV transmission area and not close to another HIV care treatment clinic experienced a positive change in enrolment after infrastructure improvements. An HIV clinic located in a limited catchment area and close to another clinic experienced negative changes in enrolment or no changes at all. Mlandizi Health Centre, an example of a new facility constructed in an area with high HIV transmission, experienced a positive change in the number of new HIV patients enrolled in care and ART. Geita District Hospital experienced a negative change in enrolment of new HIV patients because a nearby health centre started to provide ART services, which was also improved by construction of a new building to accommodate ART services.

The mean number of adult patients remaining alive and on ART increased in both rural and urban facilities after improvements. Retention rates varied between rural and urban health facilities, with urban facilities recording the highest number of patients remaining on ART after improvements. Increased access to ART during the scale-up years, greater accessibility to ART in dedicated HIV care and treatment clinics and community support organisations active in urban settings may have played a major role in retaining ART patients as compared to rural areas. The improved quality of care offered at the improved HIV care and treatment buildings may have been a factor contributing to retention in care. We did not analyse other factors related to retention in care; however, patient retention in care and alive on ART improved after PEPFAR investment in ART programmes in sub-Saharan Africa. Studies have estimated that up to 80% of patients remained in care at six month follow-up with gradual decline because of early mortality and loss to follow-up.^20,21^

The mean retention in care of children on ART increased significantly after improvements as compared to before improvements. This may be attributed to improved quality of care for children in general and increased coverage of HIV paediatric services attained after improvements. Expansion of early child HIV diagnosis and PMTCT services coupled with family-centred childcare helped children to access and remain in HIV care and treatment.^22^

The problem of attrition observed in this analysis was mostly the result of loss to follow-up and death. Overall, attrition increased after infrastructure improvements. There is no single explanation for loss to follow-up and increases in deaths of patients on ART after improvements. There is evidence that poor quality of health services and advanced disease at start of ART carries a high risk of mortality and loss to follow-up.^23,24^ We speculate that the increased loss to follow-up observed in this study was the result of unreported deaths, because most patients who started on ART during that time had severe immunodeficiency and WHO stage 3/4 in compliance with Tanzania and WHO ART guidelines, which set the threshold for initiation of ARVs at CD4+ < 200 cells/µL.^9,15^ Additional evidence shows that there are increased deaths in the first year of ART because of opportunistic infections at enrolment, stopping ART or persisting immunodeficiency.^25,26,27,28^

Limitations of our study include not being able to link patients between facilities. Patients may have been lost to follow-up in one facility but not necessarily in the district. Without specific patient level data, we were unable to analyse contributing factors leading to attrition.

## Conclusion

Patient retention in care improved in HIV care and treatment facilities with infrastructure improvements, whereas the overall number of patients newly enrolled and initiated on ART declined and attrition increased, which we suspect is partially because of patients enrolling or transferring to other facilities now closer to their homes. Conversely, health facility-specific case studies showed increases in patient enrolment in high HIV transmission areas and decreases in patient enrolment in facilities where additional HIV services were offered at nearby health facilities. Infrastructure improvements that provided adequate physical space, enhanced privacy and confidentiality, and greater accessibility to a range of HIV services may have contributed to the improved retention in care of HIV patients on ART.
